# Parasitic helminth infections of dogs, wolves, foxes, and golden jackals in Mazandaran Province, North of Iran

**DOI:** 10.14202/vetworld.2020.2643-2648

**Published:** 2020-12-12

**Authors:** Abolghasem Siyadatpanah, Abdol Sattar Pagheh, Ahmad Daryani, Shahabeddin Sarvi, Seyed Abdollah Hosseini, Roghayeh Norouzi, Larson Boundenga, Fatemeh Tabatabaie, Maria de Lourdes Pereira, Shirzad Gholami, Veeranoot Nissapatorn

**Affiliations:** 1 Ferdows School of Paramedical and Health, Birjand University of Medical Sciences, Birjand, Iran; 2 Infectious Diseases Research Center, Birjand University of Medical Sciences, Birjand, Iran; 3 Department of Medical Parasitology, Mazandaran University of Medical Sciences, Sari, Iran; 4 Department of Pathobiology, Faculty of Veterinary Medicine, University of Tabriz, Tabriz, Iran; 5 Group Evolution and Interspecies Transmission of Parasites, Department of Parasitology, Centre International de Recherches Médicales de Franceville, BP: 769, Franceville, Gabon; 6 Department of Parasitology and Mycology, Faculty of Medicine, Iran University of Medical Sciences, Tehran, Iran; 7 CICECO - Aveiro Institute of Materials and Department of Medical Sciences, University of Aveiro, Aveiro 3810, Portugal; 8 School of Allied Health Sciences and Research Excellence Center for Innovation and Health Products, Walailak University, Nakhon Si Thammarat, Thailand

**Keywords:** carnivores, environmental contamination, helminth, intestinal parasites, Iran

## Abstract

**Background and Aim:**

There is a large amount of information on intestinal parasites in stray dogs and golden jackals (*Canis aureus*) in Mazandaran Province, Iran. However, there is little information about foxes and wolves, which have a potential role in the spread of dangerous parasitic diseases, such as echinococcosis and toxocariasis. The aim of the present study was to identify the genus or species of parasitic worms in stool samples obtained from carnivores in Mazandaran Province, Iran, from August 2017 to April 2018.

**Materials and Methods:**

A total of 274 fecal samples were collected from carnivores, including dog, fox, wolf, and *C. aureus* in three areas of Mazandaran Province, Iran. All specimens were examined by centrifugal fecal flotation using a solution of Sheather’s sugar to detect helminths eggs. Then, all samples were assessed using a light microscope. Data analysis was performed by SPSS version 18 (Chicago, IL, USA).

**Results:**

In this study, seven genera of helminths were observed, including *Ancylostoma*, *Uncinaria*, *Toxocara*, *Dipylidium*, *Toxascaris*, *Taenia*, and *Spirocerca*. The prevalence of helminth infections was 97.7% (127 out of 130), 56.7% (51 out of 90), 51.4% (18 out of 35), and 52.6% (10 out of 19), among dogs, *C. aureus*, foxes, and wolves, respectively. The highest prevalence of *Ancylostoma* and *Toxocara* infections occurred in the eastern and central areas of the province (42.1% and 35.7%, respectively).

**Conclusion:**

Based on the results of this study, the infection with intestinal zoonotic helminths in carnivores was an important public health factor in Mazandaran. Therefore, these infections can be potentially harmful to humans and other animals.

## Introduction

Zoonoses are defined as diseases or ­infections that are transmitted naturally between animals and humans [1]. According to the World Health Organization, these diseases represent an important part of all newly identified infectious diseases, as well as existing ones [2]. They threaten human health because many pathogens responsible for diseases in humans are shared with other animals [3]. In fact, the emergence of these diseases among the human population is the result of increased contact between humans and animals. Therefore, several species of domestic animals, including cats, pigs, goats, and dogs, can be the reservoirs for many parasitic zoonoses.

Accordingly, the mode of transmission, namely, the direct and indirect pathways, are important for many protozoa and parasitic helminths. In addition, domestic and wild carnivores, especially stray dogs, can play a crucial role in the transmission of zoonotic helminth diseases to humans and animals in different areas of the world [4]. Canine intestinal parasites are a major concern for humans due to their presence in the marginal areas of cities and villages, especially in the cold seasons. Dogs accommodate several species of dangerous parasites responsible for various ­diseases, such as echinococcosis, ancylostomiasis, and toxocariasis. These diseases are major public health problems among the human population, especially in developing countries [5]. Although wild carnivores have an important role in natural or biological equivalence, these species are potential reservoirs of many parasites, including those that are shared between pets and humans [6]. The exchange of parasites between animals and humans can happen through the ingestion of eggs and cysts (with contaminated food, vegetables, or drinking water) or by the penetration of larvae through the skin [7,8]. The previous studies in Mazandaran and other areas of the world, including Nigeria, Australia, Belgium, and Hawaii, have shown a high rate of helminthic infections in carnivores [9-11].

The Province of Mazandaran is a region in North of Iran with suitable ecological and geographical conditions for the transmission and distribution of gastrointestinal parasites, and its change of host which may constitute a human public health problem in Mazandaran, but relatively little information on the environmental contamination by helminth eggs is currently available. Therefore, it is timely to conduct this study to investigate the possible role of wolves, dogs, foxes, and golden jackals through epidemiological screening in the transmission of parasitic helminths to other animals and humans. Furthermore, it is to explore the current trend on the epidemiology of parasitic infections by helminths of the *Canidae* family. This will further minimize exposure risk among people living in the affected areas.

## Materials and Methods

### Ethical approval

This study was approved by the Animal Ethics and Institutional Research Committee of the Mazandaran University of Medical Sciences (Ref. No: 2929).

### Study period and area

This study was conducted in Province of Mazandaran, Iran, from August 2017 to April 2018. Mazandaran is located in North of Iran and on the southeast coast of the Caspian Sea. This zone covers an area of 23,842 km^2^ with a population of about 2,922,432 individuals who live in rural and urban areas. This province has a special temperate climate and weather conditions with relative humidity of 70-100%, average temperature of 10-35°C, and annual precipitation of 800-1200 mm. This province is geographically separated into coastal plains and mountainous areas in the Alborz Mountains Range. It has various ecosystems, including grasslands, sea, and forests [5].

### Sampling

Fecal samples were collected from animals in three livestock farming zones of Mazandaran Province. A total of 274 fecal samples were obtained from *Canis familiaris*, *Vulpes vulpes*, *Canis lupus*, and *Canis aureus*. All samples were collected with the help of hunters and environmental guards. The criteria for morphological identification were the presence of parasites (shape, size, and internal structures) and confirmed with the reference standard [12], while environmental parameters were screened and confirmed by the presence of fecal matter (i.e., size, shape, and appearance), the footprints of wild carnivores and direct observation to identify the different appearances of these samples obtained from different animal species [12,13]. Approximately 6 g of each sample was placed in a small plastic bag. All the collected materials were transferred to the Parasitology Laboratory of the School of Medicine, Mazandaran University of Medical Sciences, Sari, Iran.

### Fecal examination

Briefly, approximately 3 g of feces were mixed with 10 mL of premade Sheather’s solution, with the specific gravity of 1.13, and transferred to a 15 mL conical tube. Furthermore, Sheather’s solution was added to fill in the volume to 15 mL, if required. The solution was centrifuged at 1200 rpm (280× g) for 5 min, then a coverslip (20 mm×20 mm) was added to the tube for 10 mins. The coverslip was removed, placed on a glass slide, and examined with a light microscope (Olympus BX 41TF, Tokyo, Japan) using a 10× objective and six fields were checked with a 40× objective. The morphological features and measurement methods were used to differentiate eggs from one to another [14].

## Results

**Figure-1:**
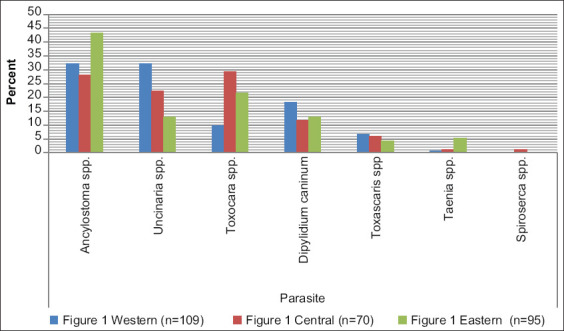
Prevalence of parasitic species found in 274 carnivorous fecal samples from three different groups.

According to the results of the current study, different genera of helminths in carnivores were distributed in different areas in Mazandaran Province, Iran. The overall prevalence of gastrointestinal parasites was 75.2%. However, the prevalence of helminthic infections was 84.4%, 82.8%, and 58.9% in the western, central, and eastern areas of the province, respectively (Figure-1).

**Figure-2:**
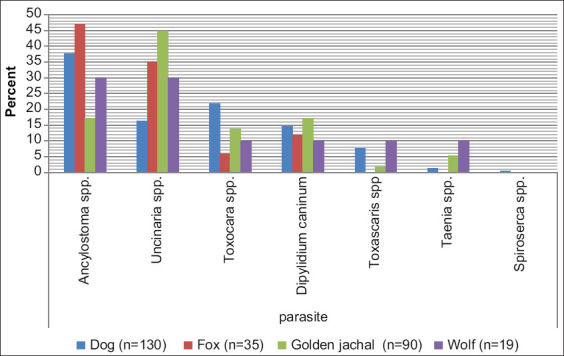
Parasites found in dogs, foxes, golden jackals, and wolf in North of Iran.

**Figure-3:**
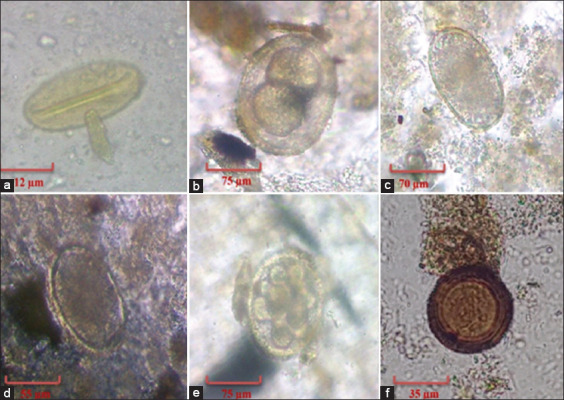
Egg of species found in carnivores in North of Iran. (a) *Spirocerca* spp., (b) immature *Toxocara* spp., (c) *Uncinaria* spp., (d) *Ancylostoma* spp., (e) *Toxocara* spp., (f) *Taenia* spp.; 400×.

Furthermore, the prevalence of helminthic infections was 97.7% (127 out of 130), 56.7% (51 out of 90), 51.4% (18 out of 35), and 52.6% (10 out of 19), among dogs, golden jackal, foxes, and wolves, respectively. The prevalence of parasites among carnivores in North of Iran is presented in Figure-2. In this study, there were seven genera of helminths, including *Ancylostoma* spp., *Uncinaria* spp., *Toxocara* spp., *Dipylidium* spp., *Toxascaris* spp., *Taenia* spp., and *Spirocerca* spp., which were examined in fecal samples from these selected animals (Figure-3).

Among these helminthic parasites, the infection rate of *Ancylostoma* spp. was found to be more prevalent in dogs (65.8%) and foxes (22.8%), while the prevalence of *Uncinaria* spp. infection was more prevalent among *C. aureus* (28.8%) and dogs (28.6%). The prevalence of *Toxocara* spp. infection was between 2.8% and 37.9%. However, dogs with an infection rate of 37.9% were the most infected animals. A higher rate of *Dipylidium caninum* infection (25.5%) was found among dogs compared to the golden jackals (11.1%). The prevalence of *Toxascaris* spp., *Taenia* spp., and *Spirocerca* spp. infections was detected between 0.7% and 13.1%. Remarkably, the highest prevalence of these parasitic infections was found among dogs with the infection rate of 13.1% with a similar spatial distribution in the province. The wolves were more infected with *Taenia* spp. (5.2%) in comparison to the dogs and golden jackals. Single and double infections were observed in 172 (62.8%) and 31 (11.3%) of carnivores, whereas poly (mixed) infections were found in 3 (1.1%) of these animals (Table-1).

**Table-1:** Mono, double, and poly (mixed) infections in examined fecal of different carnivores in North of Iran.

Number of different helminthes	Dog (n=130)		Fox (n=35)		Golden jackal (n=90)		Wolf (n=19)		Total animals	
				
	No.	%	No.	%	No.	%	No.	%	No.	%
Mono infection	100	76.9	18	51.4	44	48.9	10	52.6	172	62.8
Double infections	24	18.5	0	0	7	7.8	0	0	31	11.3
Poly infections	3	2.3	0	0	0	0	0	0	3	1.1
Total	127	97.7	17	51.4	51	56.7	10	52.6	206	75.2

## Discussion

This study attempts to demonstrate the prevalence of helminthic infections of both genus and/or genus and species of parasite worms in fecal samples from carnivores in Mazandaran Province, Iran. The overall prevalence of helminthic infections was 75.2% detected from animals in the current study. The highest prevalence of these zoonotic parasites was observed in dogs’ feces. This finding is imperative for human and domestic animals in urban and rural areas of the province. However, the zoonotic transmission of parasites from other carnivores is also crucial for humans, especially in rural areas. Our results were consistent with a previous study in Iran that reported significant infection rates of helminths in these animals. In fact, the variety in the presence of carnivore feces with different groups of helminthic parasites showed the potential contamination to all public places at any time [15,16].

In this study, two genera of *Ancylostomatidae* family were identified, including *Ancylostoma* and *Uncinaria*, which are the hookworm species that infect canines worldwide [7]. Infection with these parasites is important due to their severity and zoonotic potential [17]. *Ancylostoma* spp. and *Uncinaria* spp. cause cutaneous migration of larvae and sensitization of the gastrointestinal tract in humans, mainly in low socioeconomic conditions [18,19]. In this study, the highest prevalence rates of *Ancylostoma* spp. and *Uncinaria* spp. infections were seen in canines. However, the infection rate of *Uncinaria* spp. in dogs was almost similar to the golden jackals. The results of the present study were in accordance with a previous study in North of Iran, which found the prevalence of hookworm infections ranging from 12% to 46% [5].

*Toxocara* spp. was the second most frequent intestinal nematode, with a prevalence of 37.9% in canines. *Toxocara* spp. can spread many eggs that are resistant to environmental stresses because they have an outer shell and can remain infectious for 5 years. Therefore, their abundance in the environment can be a real health problem for the human population [7,20]. Herein, *Taenia* spp. eggs was found 5.2% of fecal samples. This finding is in accordance with the results of earlier studies in Iran [5,21]. This prevalence rate may appear low; indeed, fecal examination might underestimate the number of helminths eggs, especially those of cestode and proglottids compared to necropsy [22,23].

The overall prevalence rates of *Toxascaris* spp. in dogs, wolves, and golden jackals were 13.1%, 5.2%, and 1.1%, respectively. Surprisingly, this parasite was not detected in foxes. The presence of this parasite could be explained by the fact that dog is the natural host of this parasite. Although humans are not usually infected with this parasite, it is a cause of visceral larva migrants in children [24]. Accordingly, *Toxascaris* spp. could endanger human public health.

*D. caninum* is generally considered as a common intestinal cestode in carnivores [25]. In the present study, the prevalence of this parasitic infection was 16.7% and the highest prevalence in carnivores was observed in dogs and golden jackals, respectively. These results were inconsistent with the findings of the previous studies that reported a high prevalence of *D. caninum* in Iran (33% to 52.5%) [11] and Nigeria (75%) [26]. As a result, a high rate of this parasitic infection in dogs is not surprising. Indeed, it is a common parasite in dogs that are frequently transmitted through flea infestations [27]. However, most infected animals that are in contact with humans are asymptomatic.

*Spirocerca lupi* is the etiological agent of ­spirocercosis, a potentially fatal disease in carnivores, especially canids and *C. lupus* families [28]. This parasite is located within esophageal nodules and sheds eggs in the gastrointestinal tract and in the host’s feces. The prevalence of this parasitic infection has been reported to be 25% in Iran [29]. In our study, we found this parasite in only one dog in the central part of Mazandaran. In addition, our results were consistent with the results obtained in Latin America (Brazil and Venezuela), which reported prevalence values between 0.2% and 19% [30,31]. The presence of this parasite in dogs can be explained by the probable consumption of a paratenic host such as a lizard, hedgehog, rodent, or frog.

## Conclusion

The high prevalence rate of helminthic infections among selected carnivores in North of Iran has revealed potentially harmful effects to humans and other animals. Indeed, helminthic infections can be a plausible risk factor for public and environmental health issues. Therefore, suitable operational interventions (i.e., vaccination, neutering, and regular medical check-up) must be adopted to regulate the carnivore populations, to prevent zoonotic transmission, and to further eliminate the infection rates and disease burden. Moreover, epidemiological studies are recommended to be conducted regularly and seasonally across the country, especially in high-risk areas of Iran.

## Authors’ Contributions

AS, RN, and SG designed the study. ASP, AD, SS, SAH, LB, and FT collected the samples and analyzed the data. AS and RN drafted the manuscript. MLP and VN edited the manuscript. All authors revised and approved the final manuscript.
